# Examining The Care Pathways Patients Experience When Accessing Outpatient Psychiatric Care in British Columbia

**DOI:** 10.23889/ijpds.v9i5.2858

**Published:** 2024-09-18

**Authors:** Husayn Jessa, David Rudoler, Ruth Lavergne, Sandra Peterson, Rita McCracken, Ridhwana Kaoser

**Affiliations:** 1 University of Toronto; 2 Ontario Tech University; 3 Ontario Shores Centre for Mental Health Sciences; 4 Dalhousie University; 5 University of British Columbia; 6 Simon Fraser University

The British Columbia (BC) mental health roadmap prioritizes equitable access to care. This study investigates the equity of care pathways experienced by BC residents accessing outpatient psychiatric care for the first time. Community-based care pathways are paramount as they indicate timely access to care and are less expensive for the health system.

We analyzed linked administrative BC health data spanning April 1st 2013, to March 31st 2022, to study how sociodemographic factors relate to accessing psychiatric care pathways. Pathways to outpatient psychiatric care include a community-based pathway, hospitalization to primary care to outpatient psychiatry, and hospitalization directly to outpatient psychiatry. Sociodemographic factors considered were administrative sex, immigration recency and status, neighbourhood income quintile and being a recipient of a low-income drug plan. These relationships were analyzed using multinomial logistic regression, adjusting for age, location, fiscal year, and stratifying by mental disorder diagnoses.

Being male, a low-income drug plan recipient, living in a lower income neighbourhood, or being a recent economic immigrant or permit holder was associated with increased odds of accessing outpatient psychiatric care via either hospitalization pathway. Having a diagnosis of schizophrenia/bipolar disorder was independently associated with both hospitalization pathways, and this diagnosis exacerbated the magnitude of the estimates for the sociodemographic characteristics.

We find clear disparities in pathways to accessing outpatient psychiatric care. These disparities indicate health system failures in addressing specific population needs, highlighting the importance of targeted interventions, such as increasing the number of community health workers, to achieve equitable mental health care in BC.

